# An approach for comparing agricultural development to societal visions

**DOI:** 10.1007/s13593-021-00739-3

**Published:** 2022-01-13

**Authors:** Julian Helfenstein, Vasco Diogo, Matthias Bürgi, Peter H. Verburg, Beatrice Schüpbach, Erich Szerencsits, Franziska Mohr, Michael Siegrist, Rebecca Swart, Felix Herzog

**Affiliations:** 1 Agroecology and Environment, Agroscope, Zürich, Switzerland; 2 Land Change Science Research Unit, Swiss Federal Research Institute WSL, 8903 Birmensdorf, Switzerland; 3 Institute of Geography, University of Bern, Bern, Switzerland; 4 Environmental Geography Group, Institute for Environmental Studies (IVM), Vrije Universiteit Amsterdam, 1081 HV Amsterdam, The Netherlands; 5 Department of Health Sciences and Technology, ETH Zürich, Zürich, Switzerland

**Keywords:** Sustainable intensification, Sustainable agriculture, Sustainability assessment, Farming, Food system, Normative scenario, Agricultural landscape, Farm interview, Agricultural intensity

## Abstract

There is broad agreement that agriculture has to become more sustainable in order to provide enough affordable, healthy food at minimal environmental and social costs. But what is “more sustainable”? More often than not, different stakeholders have opposing opinions on what a more sustainable future should look like. This normative dimension is rarely explicitly addressed in sustainability assessments. In this study, we present an approach to assess the sustainability of agricultural development that explicitly accounts for the normative dimension by comparing observed development with various societal visions. We illustrate the approach by analyzing farm- and landscape-scale development as well as sustainability outcomes in a Swiss case study landscape. Observed changes were juxtaposed with desired changes by Avenir Suisse, a liberal think tank representing free-market interests; the Swiss Farmers Association, representing a conservative force; and Landwirtschaft mit Zukunft, an exponent of the Swiss agroecological movement. Overall, the observed developments aligned most closely with desired developments of the liberal think-tank (72%). Farmer interviews revealed that in the case study area farms increased in size (+ 57%) and became more specialized and more productive (+ 223%) over the past 20 years. In addition, interpretation of aerial photographs indicated that farming became more rationalized at the landscape level, with increasing field sizes (+ 34%) and removal of solitary field trees (− 18%). The case study example highlights the varying degrees to which current developments in agriculture align with societal visions. By using societal visions as benchmarks to track the progress of agricultural development, while explicitly addressing their narratives and respective systems of values and norms, this approach offers opportunities to inform also the wider public on the extent to which current developments are consistent with different visions. This could help identify mismatches between desired and actual development and pave the way for designing new policies.

**Supplementary Information:**

The online version contains supplementary material available at 10.1007/s13593-021-00739-3.

## Introduction

Farmers, consumers, and policymakers largely agree that agriculture has to become more sustainable, yet there is rarely consensus on what a sustainable future should look like. Different stakeholders have opposing opinions on how impacts and tradeoffs between different sustainability dimensions should be weighted (Robinson et al. 2011; Zorondo-Rodríguez et al. 2014). Based on these different perceptions, some argue that digitalization, precision farming, and automatization are the solution (Walter et al. 2017; Wolfert et al. 2017), while others promote a decrease in management intensity and the adoption of ecological principles in farming (Altieri 1995; Wezel et al. 2020). Even ecologists are divided between the vision to intensify existing agricultural land while leaving as much land aside for conservation (land sparing) and the vision to implement less-intensive agriculture but on more land (land sharing) (Phalan et al. 2011; Fischer et al. 2011). These inherent but often not explicitly declared values have been shown to bias individual sustainability assessment tools (Svarstad et al. 2008; Binder et al. 2010; Chopin et al. 2021), challenging the legitimacy of sustainability research (van der Hel 2018). Such biases are especially relevant when assessing complex agricultural systems, which require accounting for multiple objectives playing out across a variety of spatial and temporal scales (Helfenstein et al. 2020). While calls are growing louder to acknowledge that stakeholders have conflicting interests, norms, expectations, and visions in sustainability assessments (Miller et al. 2014; Pascual et al. 2017; Schlaile et al. 2017; Chopin et al. 2021), a systematic approach that reconciles different value attributions and weightings of sustainability assessment categories is missing.

Holistic sustainability assessments and mapping the perceptions of different stakeholder groups are common in addressing sustainability in participatory integrated assessments of e.g. land-use change or water management (Ridder and Pahl-Wostl 2005; Morris et al. 2011; König et al. 2013); however, they are still rare in assessments of agricultural sustainability. In a review of over 100 tools available for farm sustainability assessment, only 14% qualified as having stakeholder participation (Chopin et al. 2021). Most sustainability assessments continue to measure farming system performance by comparing indicator values with reference values, with little or no stakeholder involvement in defining sustainability objectives (Binder et al. 2010; Schader et al. 2014). Thus, Chopin et al. (2021) highlight the need for new temporally dynamic approaches for assessing farm sustainability and accounting for stakeholder sustainability framing. While earlier work considered a multiscale approach, accounting for sustainability outcomes at the farm as well as the landscape scale and over several years (Chopin et al. 2017), such a multiscale approach has not yet been linked to accommodate sustainability objectives of various stakeholder groups.

A possible approach to accommodate multiple objectives and interpretations of sustainability is to juxtapose observed or projected development with political and societal visions, also called normative scenarios (Rounsevell and Metzger 2010). Scenarios are “plausible and often simplified descriptions of how the future may develop, based on a coherent and internally consistent set of assumptions about driving forces and key relationships” (IPCC 2014). Visions, on the other hand, are common understandings of a stakeholder group for the desired future (Pérez-Soba et al. 2018). Hence, visions are a subgroup of scenarios that focus on what should be. Visions are a powerful tool to stimulate dialogue, for example, the biodiversity futures for reaching biodiversity targets (Wyborn et al. 2020) or, more broadly, the Global Scenario Group’s multiple visions for world development in the twenty-first century (Electris et al. 2009). Also, visions can be linked to observed or projected, development to identify mismatches between projected and desired states, laying the foundation for corrective policies (Pérez-Soba et al. 2018; Verkerk et al. 2018). Since societal visions reflect societal interests, such an approach is able to accommodate a broad range of different, but equally legitimate sustainability objectives and concerns. As such, using visions as benchmarks for measured developments, especially if the visions transparently convey interests from multiple stakeholder groups, can increase legitimacy compared to approaches proposing an (absolute) preconception of sustainability (Rounsevell and Metzger 2010; Kenter et al. 2015). While earlier work has developed future scenarios for agriculture in a participative manner (Mitter et al. 2020), societal visions have not yet been used as benchmarks to assess current agricultural development.

**Fig. 1:**
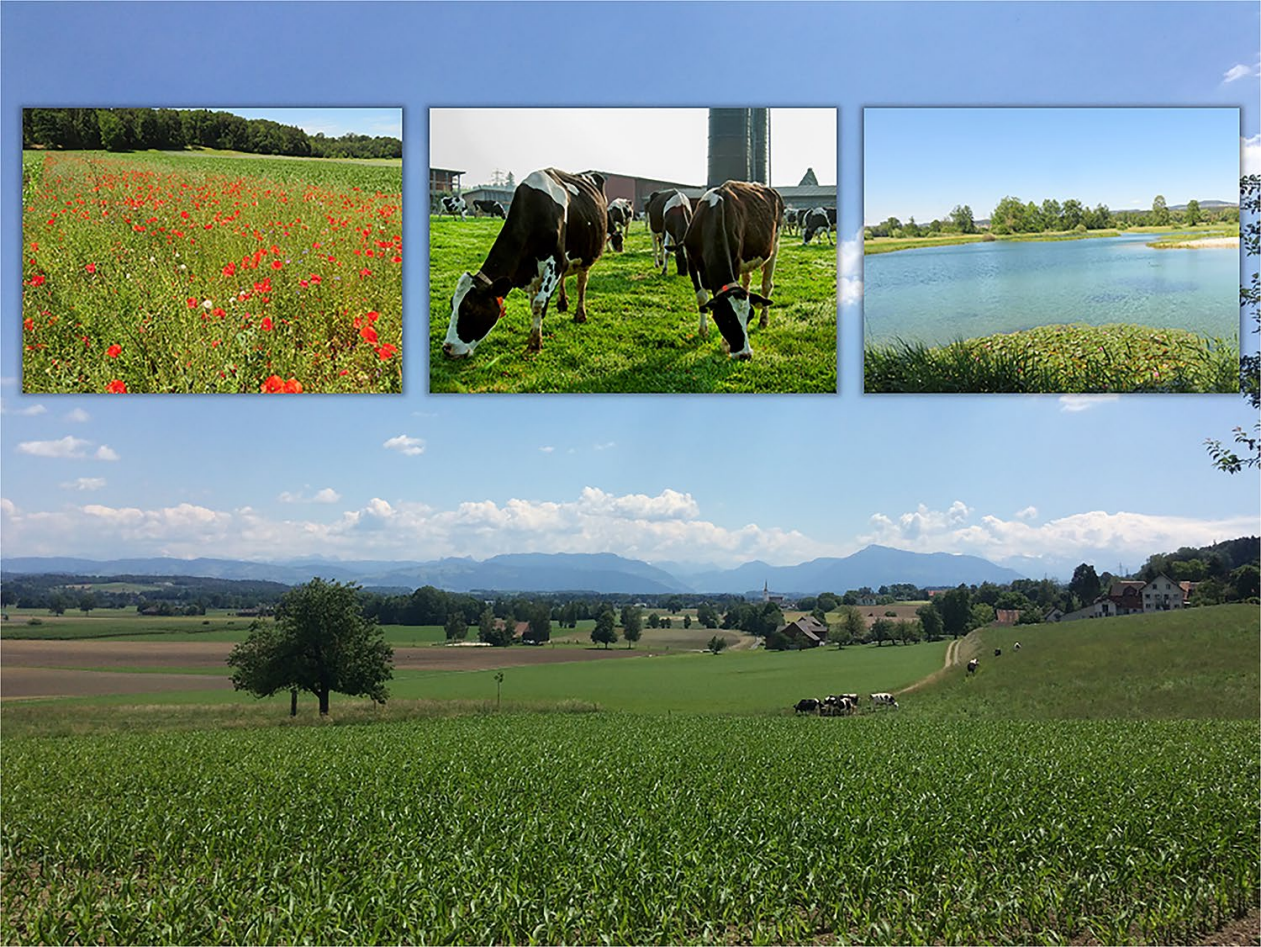
Reusstal, a typical Swiss lowland agricultural landscape. The landscape consists of a mosaic of intensive agricultural areas for crop and livestock production, settlements, and wetland conservation areas. Photographs by Erich Szerencsits, Gabriela Brändle, and Franziska Mohr.

The objective of this paper is to present a systematic approach that accounts for different sustainability value systems when assessing the development of agricultural landscapes. We test and illustrate the approach by assessing the agricultural development over the past twenty years in a typical Swiss lowland agricultural landscape (Fig. 1) while pursuing the following research questions:

- How has agriculture developed over the past 20 years regarding the three aspects of sustainability: ecological impact, the economic performance of the farms, and the social well-being of the farmers?
- How do these changes align with societal visions for future agricultural systems?

To meet these objectives, we described the trajectory of 22 different indicators covering farm and landscape-scale development as well as social, economic, and environmental aspects of sustainability. The observed developments are then contrasted with visions of three conflicting exponents of agricultural politics in Switzerland to determine the agreement between observed and desired change.

## Methods

### Approach for comparing current developments to normative visions

**Fig. 2:**
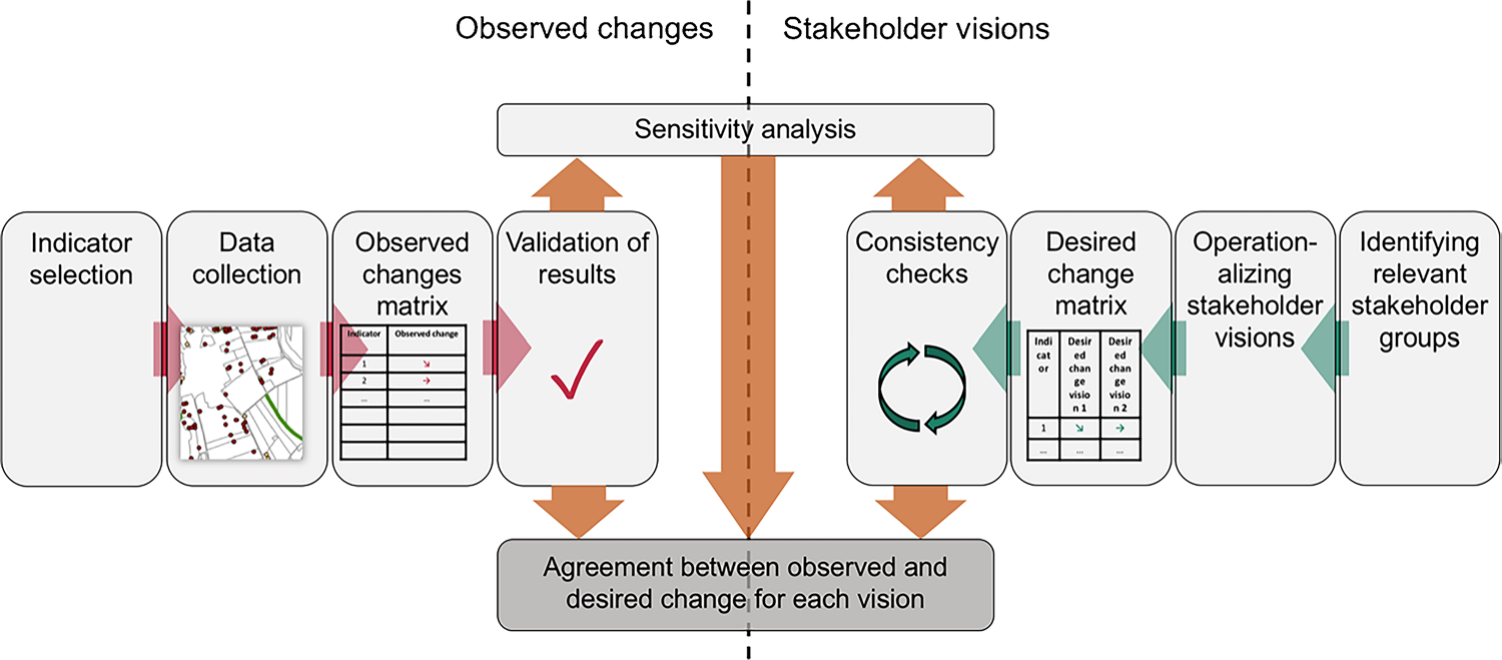
Overview of the approach to confronting observed changes with stakeholder visions. The individual steps are described in the Methods section.

Agricultural development was assessed using farmer interviews and landscape mapping (Fig. 2, left). This part of the assessment included indicator selection, data collection, data analysis to create an observed change matrix, and validation of the results. We then confronted the observed changes with conflicting societal visions. This was done by identifying stakeholder groups driving agricultural development at the national level, extracting desired changes from grey literature describing the stakeholder visions, and checking the consistency of the resulting desired change matrix (Fig. 2, right). Finally, we performed a sensitivity analysis and calculated the agreement between observed and desired change for each vision.

### Case study site

The Reusstal between Rottenschwil and Mühlau is a typical Swiss lowland agricultural landscape with residential areas, intensive agriculture, and nature conservation in close proximity (Fig. 1). The case study site covers 25 km^2^ and stretches along the river Reuss with no noteworthy altitude or topographical heterogeneity, spanning the municipalities Rottenschwil, Aristau, Merenschwand, Mühlau, and the northern part of Hünenberg. While historically, these lowlands were swampy and only extensively used as grazing grounds, the river Reuss was channeled, and most of the surrounding swamps drained in the 1970s, making intensive agriculture possible (Kanton Aargau 1982). The hills and the areas around the settlements were traditionally used as silvopastoral systems with high-stem fruit trees (Streuobstwiesen), but these trees have been declining since the 1950s (Herzog 2000). The population continues to grow considerably in this area due to job opportunities in nearby cities (+ 12% from 2010 to 2018 in the largest municipality of Merenschwand), putting increasing pressure on agricultural land (BFS 2020). Conservation areas of national importance are scattered throughout the study area, including a wetland area (Maschwander Allmend) and two riparian areas (Still Rüss-Rickenbach and Ober Schachen-Rüssspitz) (BAFU 2017a, b, c). In 2002, the habitats in the study area were mapped and management intensity interviews were conducted with farmers as part of a pan-European study that explored relationships in the triangle of landscape structure, land-use intensity, and their effects on biodiversity (Herzog et al. 2006; Hendrickx et al. 2007; Billeter et al. 2008). From this earlier work, it was known that most farms in the area specialize in dairy or pig production mixed with arable cropping, while some farms also produce field vegetables.

### Farmer interviews

We performed structured face-to-face interviews with 20 randomly selected farmers in the study region to determine changes in crop and livestock management, as well as impacts on sustainability. From a list of all farmers from the four main municipalities (Rottenschwil, Aristau, Merenschwand, and Mühlau) with at least 5 ha of land and 80% of their land within the study perimeter (*n* = 48), we randomly contacted farmers until we obtained 20 interviews. The response rate, calculated as the number of farmers agreeing to an interview divided by the number of farmers reached, was 71% (see Section 3.3 for the spatial coverage of the farms). Questions in the interviews probed general farm characteristics, livestock production, arable and permanent crop production, as well as social, economic, and environmental impacts. This included a detailed listing of crops grown and livestock held on the farm. Each question had two parts. First, farmers were asked how the situation is today (e.g., how much agricultural land is managed by the farm?). Second, farmers were asked how the current situation compares to the situation 20 years ago (e.g., how much agricultural land was managed by the farm 20 years ago?). If farmers were unsure, they could answer, “don’t know.” The complete questionnaire can be found in the supplement.

A preliminary version of the questionnaire was tested in Reusstal in March 2020. Based on these experiences, the questionnaire was revised, and farmers were interviewed with the final version in September and October 2020. All interviewees provided their informed written consent. The experimental design and the questionnaires received ethical clearance from the Ethical Commission of the Swiss Federal Institute of Technology (ETH-EK 2020-N-146).

### Landscape mapping

We visually interpreted aerial orthophotos from 1998, 2012, and 2017 to determine changes in land use and landscape structure (Swisstopo 2017). The spatial resolution of the images was 50, 25, and 10 cm, respectively. Tree rows and hedges were mapped as linear habitat types. Field trees were mapped as point elements, divided into small (2–5 m canopy diameter) and large (> 5 m canopy diameter) trees. A description of the habitat types and their respective qualifiers can be found in the supplement (Supplementary Table 1). The minimal mapping unit was 25 m^2^ for areal elements and 40 m for linear elements.

Land cover was classified following the European Nature Information System (EUNIS) habitat classification (EEA 2019). We mapped the broadest habitat classes from EUNIS known to occur in the study site (EUNIS code in parentheses): settlement (J), barren land (H), water (C), forest (G), wetlands (D), grasslands (E), crops (I), shrub plantations (FB), and orchards (G1.D) (Supplementary Table 1). While EUNIS has a habitat focus, our study focused on agricultural land-use intensity. Hence, we added levels of land-use intensity to several EUNIS classes. Firstly, grassland was further divided into intensive grassland (grassland mown or grazed more than 3 times per year) and extensive grassland and other extensively managed agricultural areas such as flower strips and fallows. While flower strips and field margin vegetation could be identified with high certainty from orthophotos, it was sometimes difficult to differentiate between extensive grasslands and intensive grasslands, especially for the older images. In such cases, other orthophotos from about the same year were consulted, which may show different phenological stages. Wetlands could be distinguished from extensive or intensive grasslands by complementing orthophoto interpretation with wetland signatures on topographical maps. If all previous approaches were inconclusive, an expert decision was made based on the authors’ knowledge of the study area (several authors of the study have been conducting research in the area for 20 years or more). Secondly, orchards were divided into intensive fruit production and high-stem orchards. Intensive orchards were defined as low-stem, which appear as closed rows rather than an assortment of individual trees on orthophotos. High-stem orchards were defined as areas with at least three field trees and a tree density of  more than 20 trees ha^−1^ (Herzog 2000), which was calculated based on the number of mapped trees and parcel boundaries.

### Indicators to characterize agricultural development

We analyzed agricultural development both at the farm and the landscape scale as well as changes in social, economic, and environmental dimensions of sustainability (Helfenstein et al. 2020). Farm-scale development was measured with indicators on farm area, livestock units, crop diversity, livestock diversity, and feed import (Table 1). These five indicators cover important processes of farm size growth and specialization, which are two key drivers of agricultural intensification (van Vliet et al. 2015). At the landscape level, we chose to quantify agricultural field size, since this is an indicator of land management intensity with implications for biodiversity (Geiger et al. 2010; Clough et al. 2020), as well as total agricultural area and proportion of intensively used agricultural land (Billeter et al. 2008).

**Table. 1:** Indicators used to characterize agricultural development. We differentiated between indicators related to farm-scale development, landscape-scale development as well as social, economic, and environmental outcomes. GIS geographic information system.

Indicator	Unit	Definition	Method of assessment	Spatial scale
Farm-scale development				
Farm area	ha	Agricultural area managed by farm	Interviews	Farm
Livestock units	LU	Livestock units per farm using national livestock unit conversion factors (Agridea 2019)	Interviews	Farm
Crop diversity	Count	Number of crops cultivated per farm	Interviews	Farm
Livestock diversity	Count	Number of livestock categories held per farm	Interviews	Farm
Feed import	%	Percentage of livestock feed purchased from retailer	Interviews	Farm
Landscape-scale development				
Average field size	ha	Average size of crops, intensive grassland, and intensive orchard polygons	GIS	Landscape
Total agricultural area	ha	Total agricultural area in study area	GIS	Landscape
Proportion of intensively used agricultural land	%	Percentage of intensively used agricultural area (crops, intensive grassland, and intensive orchard) in relation to total study area	GIS	Landscape
Social				
Farmer satisfaction	Likert-scale	Farmer's reported satisfaction with his/her work on the farm	Interviews	Farm
Societal valuation	Likert-scale	Farmer's perceived societal valuation of his/her work on the farm	Interviews	Farm
Fraction of farmers > 50 years old	%	Percentage of farm holders over 50 years old	Interviews	Landscape
Successor	%	Percentage of farmers over 55 with a defined successor	Interviews	Farm
Fraction of own land	%	Percentage of owned as opposed to leased land	Interviews	Farm
Economic				
Farm economic situation	Likert-scale	Farmer's perceived economic situation of the farm	Interviews	Farm
Price trend	%	Change in price received for the most important agricultural product	Interviews	Farm
Production trend	%	Change in production volume of the most important product	Interviews	Farm
Off-farm work	%	Percentage of income generated by off-farm work	Interviews	Farm
Environmental				
Ecological focus area	%	Percentage of farm area qualified for agri-environment scheme direct payments	Interviews	Farm
Semi-natural habitats	%	Percentage of landscape covered by semi-natural habitat (here wetlands, extensively managed lands, high-stem orchards, and forest)	GIS	Landscape
N intensity	kg N ha^−1^	N fertilizer application from all sources on main crop	Interviews	Farm
Pesticide use	count	Number of pesticide applications on main crop	Interviews	Farm
Livestock density	LU ha^−1^	Livestock units per agricultural area	Interviews	Farm

Well-being, both of farmers and surrounding communities, is considered a key, yet it is difficult to measure an aspect of social sustainability (Janker and Mann 2018). To determine farmer well-being, we asked farmers to rate their general satisfaction with their work, and if satisfaction has deteriorated, stayed the same, or improved. Similarly, we asked farmers to rate perceived societal valuation for their work on the farm, and if it has deteriorated, stayed the same, or improved. The assumption was that if the pursued farm strategy has positive impacts on community well-being, the farmers would experience improved societal valuation and vice versa. We focused on farmer aging and succession as the second key aspect of social sustainability to address the problem of an aging farmer population in Europe (Potter and Lobley 1992; Micha et al. 2020). The indicator for farmer age was the percentage of farmers over 50 years old compared to the national average of 56% (Erdin 2017). The share of farmers with a successor was only determined for older farmers (> 55) (Fischer and Burton 2014). Finally, we calculated the fraction of own as opposed to the leased land.

Economic sustainability was measured by asking farmers about the general economic situation, price trend, and production trend. Farmers were asked to rate the general economic situation of the farm, and if it has deteriorated, stayed the same, or improved. In addition, we asked farmers about the relative change in price received for their most important product since 2000 and the change in production volume over the same period. Also, we asked farmers what percentage of their income they earn through off-farm work (Table 1).

Environmental sustainability was assessed by measuring habitat quantity as well as environmentally relevant farm emissions. Habitat quantity was assessed both at the farm scale (ecological focus area) and at the landscape scale (semi-natural habitats). The ecological focus area was defined as the fraction of farm area eligible for agri-environment scheme direct payments and probed in interviews (Table 1). The share of semi-natural habitat cover was defined as the area of high-stem orchards, extensively managed areas, wetlands, and forests, thereby including not only agricultural land but total semi-natural habitat cover at the landscape scale (Herzog et al. 2017). Since fertilizer and pesticide applications vary from crop to crop, we asked farmers about changes in applications to their main crop (Herzog et al. 2006; Geiger et al. 2010). Finally, we calculated livestock density based on farm area and livestock units per farm as a proxy for livestock-related greenhouse gas and nutrient emissions (Herzog et al. 2006). While the environmental indicators used in this study do not measure actual outcomes (e.g., loss of biodiversity, water pollution, greenhouse gas emissions), a reduction/increase in the magnitude of these indicators has been shown to improve/reduce environmental sustainability dimensions (Hendrickx et al. 2007; Geiger et al. 2010; Herzog et al. 2017).

### Statistical analysis and validation of observed changes

We used a Wilcoxon signed-rank test for paired samples on interview data with numerical answers to determine significant changes in answers reported for 2020 versus the answers reported for 2000. We used a Wilcoxon signed-rank test rather than a Student’s *t*-test since the former does not assume normally distributed samples. The difference in average field size between 1998 and 2017 was also assessed by the Wilcoxon test, but for unpaired samples. Significant differences were determined at *p* < 0.05.

We validated the consistency of our results through the process of triangulation (comparing results from different sources). Firstly, some of the questionnaire answers and mapping results regarding past land use could be compared to farmer surveys and in-field mapping performed 20 years ago in the same study region (Herzog et al. 2006; Bailey et al. 2007). Secondly, we compared farmer-reported land use with landscape mapping results to cross-validate the two approaches.

### Stakeholder groups and visions

A recent analysis of actors in Swiss agricultural policy identified three main clusters in terms of belief similarity: a liberal cluster, a conservative cluster, and a green cluster (Metz et al. 2020). The conservative cluster has a consistently higher preference for domestic support of agricultural production than the green and the liberal clusters. Regarding agricultural “greening,” there is most support from the green cluster, followed by the liberal cluster, and least support by the conservative cluster (Metz et al. 2020). In our study, we selected extreme representatives from each of these three clusters that have articulated clear visions for a more sustainable future of agriculture: Avenir Suisse (AS), the Swiss Farmer’s Association (SFA), and Landwirtschaft mit Zukunft (LmZ). These three organizations embody conflicting societal visions for agriculture in Switzerland. Avenir Suisse is a liberal think tank that promotes reducing dependency on agricultural subsidies through increasing resource use efficiency, more free trade, and a transition to larger, more specialized, and more globally-competitive farms (Dümmler and Roten 2018). They argue that farming should be concentrated on activities and regions where it can be competitive, so that more land is available to pursue other goals, from development to biodiversity conservation (Dümmler and Anthamatten 2020). The Swiss Farmer’s Association, on the other hand, represents the interests of farmers in Switzerland, with the main goal to retain current forms of production while improving farmer well-being and income (Monin et al. 2018). The Swiss Farmers’ Association is the most conservative of the actors studied in Metz et al. (2020). Landwirtschaft mit Zukunft is a recently founded umbrella organization of the agroecological movement that represents many environmental organizations including Greenpeace, World Wildlife Fund, Smallholder Association, and Swiss Organic Farmers (Kehnel et al. 2018). They support a transformative “greening” of agriculture through smaller, more diversified farms with less livestock and higher levels of biodiversity.

We summarized the visions of each of the three stakeholders in relation to the indicators used in this study in the desired change matrix. The desired change matrix was completed by screening available literature outlining the visions of the three stakeholders. More precisely, we based desired change on the 10-point strategy document (Dümmler and Roten 2018) as well as an update from 2020 (Dümmler and Anthamatten 2020) from Avenir Suisse, the strategic document of the Swiss Farmers Association outlining their vision until 2050 (Monin et al. 2018), and the Vision for Agriculture in 2030 from Landwirtschaft mit Zukunft (Kehnel et al. 2018). For each indicator, we reported whether an increase (+ 1), no change (0), or decrease (− 1) was desired by each vision according to the goals outlined in these documents. In addition, we defined weights for each vision and indicator pair based on the importance of the desired change for the respective vision. If the indicator was mentioned or implied but not central to the respective vision, the desired change was weighed with (1). If the desired change was central to the respective vision, a double weight (2) was given. If the indicator was not mentioned or implied, a weight of 0 was given to indicate that this indicator was not important for the respective stakeholder vision. Since the screened documents describing each vision did not contain explicit mentions of weights, the weight assignments represent a key assumption in the analysis. We checked the consistency of the desired change matrix by comparing desired change with actor beliefs and preferences, as analyzed in Metz et al. (2020).

### Agreement between observed and desired change

To link observed developments with stakeholder visions, we determined to what degree actual change overlapped with stakeholders’ desired change following an approach similar to the one proposed by Verkerk et al. (2018). In a first step, observed change of both qualitative and quantitative indicators was reclassified into three classes for each farm (Verkerk et al. 2018). Increase corresponded to + 1, no change to 0, and decrease to − 1. For numerical variables, we assumed a threshold of 5% to determine whether an indicator had changed (Verkerk et al. 2018). Since the no-change threshold is critical for calculating agreement between observed and desired change, this threshold represents a second key assumption in the analysis. Landscape-level indicators (e.g., total agricultural area) could not be calculated at the farm level, and each farm received the same (landscape average) value.

Second, for each farm, we compared the observed change with the desired change by calculating the absolute difference between reclassified observed change and the desired change overall or over a subset of indicators (*n*) (Eq. 1):

1 $$\mathrm{Difference between observed and desired change} = \sum_{i=1}^{n}\mathrm{abs}\left({o}_{i}-{d}_{i}\right)*{w}_{i}$$

where *o*_*i*_ is the reclassified observed change, *d*_*i*_ is the desired change, and *w*_*i*_ is the respective weight. For each farm and any *n* number of indicators, potential agreement with a given vision was calculated as the sum of the weights times two (Eq. 2):

2 $$\mathrm{Potential agreement}={\textstyle\sum_{i=1}^n}\;2\ast w_i$$

The actual agreement over all or a subset of indicators (*n*) was then calculated as the difference between *potential agreement* and the *difference between observed and desired change* divided by *potential *agreement (Eq. 3). By calculating agreement relative to potential agreement, % agreement is independent of the number of indicators and weights and can be used to compare different visions. Zero agreement would imply that none of the observed changes was in line with the respective vision, whereas 100% agreement would imply that all changes were in line with the respective vision.

3 $$\mathrm{Agreement}=100*\frac{\left(\mathrm{potential agreement}-\mathrm{difference between observed and desired change}\right)}{\mathrm{potential agreement}}$$

A Kruskal-Wallis one-way analysis of variance, followed by post hoc Dunn-test with Benjamini–Hochberg adjustment (Benjamini and Hochberg 1995), was used to test whether there was a statistical difference between agreements with different visions. Finally, we performed a sensitivity analysis to determine the influence of two key assumptions (weights in the desired change matrix and the “no change” threshold discussed above) on the calculated agreement. To test the sensitivity of the results on the distribution of weights, we re-ran the analysis with equal weights (all weights = 1). To test the sensitivity of the results on the “no change” threshold, we conducted the analysis while varying the “no change” threshold from 0 to 10% by 1% increments.

## Results and discussion

### Farm-scale development and sustainability outcomes

Farmer interviews revealed considerable changes in farm structure and management over the past twenty years (Fig. 2a). The average farm size increased from 24 to 38 ha (Wilcoxon test, *p* < 0.01) (Table 2). Concomitantly, the average total livestock units per farm increased from 42 to 69 (*p* < 0.01). The most important crops by area were corn, wheat, carrots, and potatoes. While 14 different crops were reported, 33% of the crop area was corn, and corn was the most important crop by area for 75% of farmers. There was no significant trend in crop diversity over time (Table 2). All but two farmers had livestock, mostly dairy cows, pigs, beef cattle, and poultry. Eight farms were classified as dairy farms, ten as mixed (arable with dairy, cattle, and/or pig), and two were purely arable with no livestock. Three farmers reported quitting dairy during the period under study. Livestock diversity decreased from on average 5.1 different livestock types per farm in 2000 to 3.7 in 2020 (*p* = 0.02), as farms specialized more on certain livestock types and products. The reported share of animal feed purchased from retailers (feed import) was on average 20% in 2000 and did not change significantly over time (Supplementary Fig. 1a).

**Table. 2:** Agricultural development over the past two decades based on 24 indicators. Columns 2000 and 2020 show the mean ± the standard deviation for the period of analysis. For landscape-level indicators there are no replicates, so only means are reported. For qualitative indicators, the general trend is shown with arrows. For all interview-based indicators, *n* shows the sample size. ^a^Assessed at the landscape-level (only one value); ^b^qualitative indicator.

Indicator	2000	2020	Wilcoxon test, *p*-value	*n*
Farm-scale development				
Farm area [ha]	23.9 ± 7.9	37.7 ± 27.0	< 0.01	20
Livestock units (LU)	42.3 ± 24.5	69.2 ± 51.0	< 0.01	20
Crop diversity	3.2 ± 1.2	3.0 ± 1.8	0.42	18
Livestock diversity	5.1 ± 1.8	3.7 ± 1.8	0.02	19
Feed import [%]	20 ± 21	26 ± 24	0.47	16
Landscape-scale development				
Average field size [ha]	1.35 ± 1.25	1.81 ± 1.56	< 0.001	-
Total agricultural area [ha]^a^	1781	1737	-	-
Proportion of intensively used agricultural land [%]^a^	93	90	-	-
Social				
Farmer satisfaction^b^	-	→	-	18
Societal valuation^b^	-	↘	-	18
Fraction of farmers over 50 years old [%]^a^	-	35	-	20
Successor [%]^a^	-	67	-	20
Fraction of owned land [%]	68 ± 24	57 ± 25	0.04	20
Economic				
Farm economic situation^b^	-	→	-	20
Price trend [% of 2000 price]	-	-14.3 ± 13.5	-	20
Production trend [% of 2000 volume]	-	223 ± 283	-	20
Off-farm work [%]	13.7 ± 25.9	22.8 ± 31.7	0.18	19
Environmental				
Ecological focus area [% of farm area]	12 ± 7	18 ± 10	< 0.01	18
Semi-natural habitats [% of landscape area]^a^	19.8	22.2	-	-
N-intensity [kg N ha^−1^]	132 ± 21	133 ± 21	1.00	14
Pesticide use [number of applications]	2.0 ± 1.9	2.1 ± 2.3	0.71	20
Livestock density [LU ha^–1^]	1.9 ± 0.8	2.1 ± 1.1	0.30	20

Farmer satisfaction was stable, with about the same number of farmers reporting a decrease in satisfaction as an increase (Supplementary Fig. 1b). However, 61% of respondents reported that perceived societal valuation had decreased, which farmers explained in conversation was related to growing political and societal pressure to reduce fertilizer and pesticide use. With only 35% of farmers over 50 years old (compared to the national average of 56%, Erdin et al. 2017), the sample does not show an over-aging of farmers in the study region. Of the farmers interviewed who were over 55 years old, 67% had a successor. The fraction of own land as opposed to leased land decreased during the period of study (*p* = 0.04) (Table 2).

Overall, there was no clear trend in the perceived economic situation (Supplementary Fig. 1b), but there are more pronounced trends for individual farm types: while 50% of dairy farmers reported being worse off today than 20 years ago, this value was only 25% for non-dairy farmers. Deteriorating economic situations for dairy farms was strongly related to a falling milk price, which was reported to be 25% lower today than 20 years ago. Non-dairy farmers reported no or only small changes in prices received for their main products (Table 2). All farmers either increased (82%) or maintained (18%) production volumes of their most important product (Supplementary Fig. 1a). On average reported production per farm more than doubled (Table 2). The high gains in productivity were mostly the result of farm growth and specialization (e.g., increasing animal numbers while outsourcing parts of their lifecycle to other farms), and only to a lesser degree due to gains in efficiency (e.g., higher milk output per input). Most farmers worked full time on the farm. While off-farm employment tended to increase, there was a large spread in the data and thus no significant effect (Table 2).

The average share of ecological focus areas for biodiversity promotion increased from 12 to 18% of agricultural land per farm (*p* < 0.01). The average N intensity on the main crop was around 130 kg N ha^−1^ and did not change during the study period. There was a large scatter in the number of pesticide applications related to crop types. While farmers whose main crop was corn reported applying just one herbicide application, farmers whose main crop was rapeseed or carrots had up to 10 pesticide applications per growing season. However, there was no trend in the number of pesticide applications over time (Fig. 2a). Since the average farm area increased in parallel to livestock units, livestock density also did not change significantly over time (Table 2).

### Landscape-scale agricultural development

**Fig. 3:**
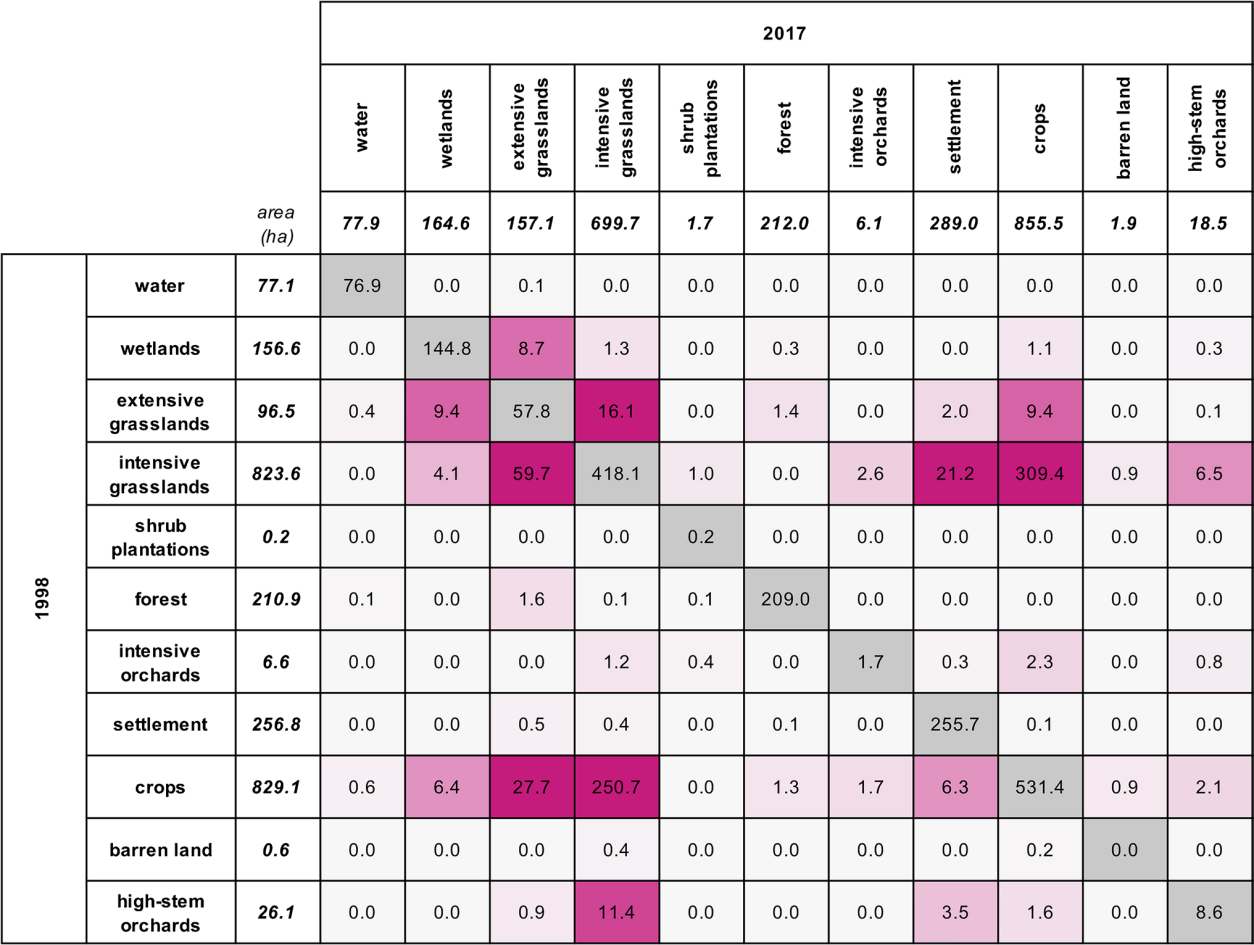
Land-use change matrix. The matrix shows how much of each land use was converted to another land use from 1998 to 2017. The diagonal (shaded gray) corresponds to the area of each land use that stayed persistent. The darker the shade of pink, the larger the change. Change from intensive grassland to crop and vice versa is not informative because intensive grassland is part of the crop rotation. Extensive grassland includes field margin vegetation, flower strips, and other extensively managed agricultural lands.

**Fig. 4:**
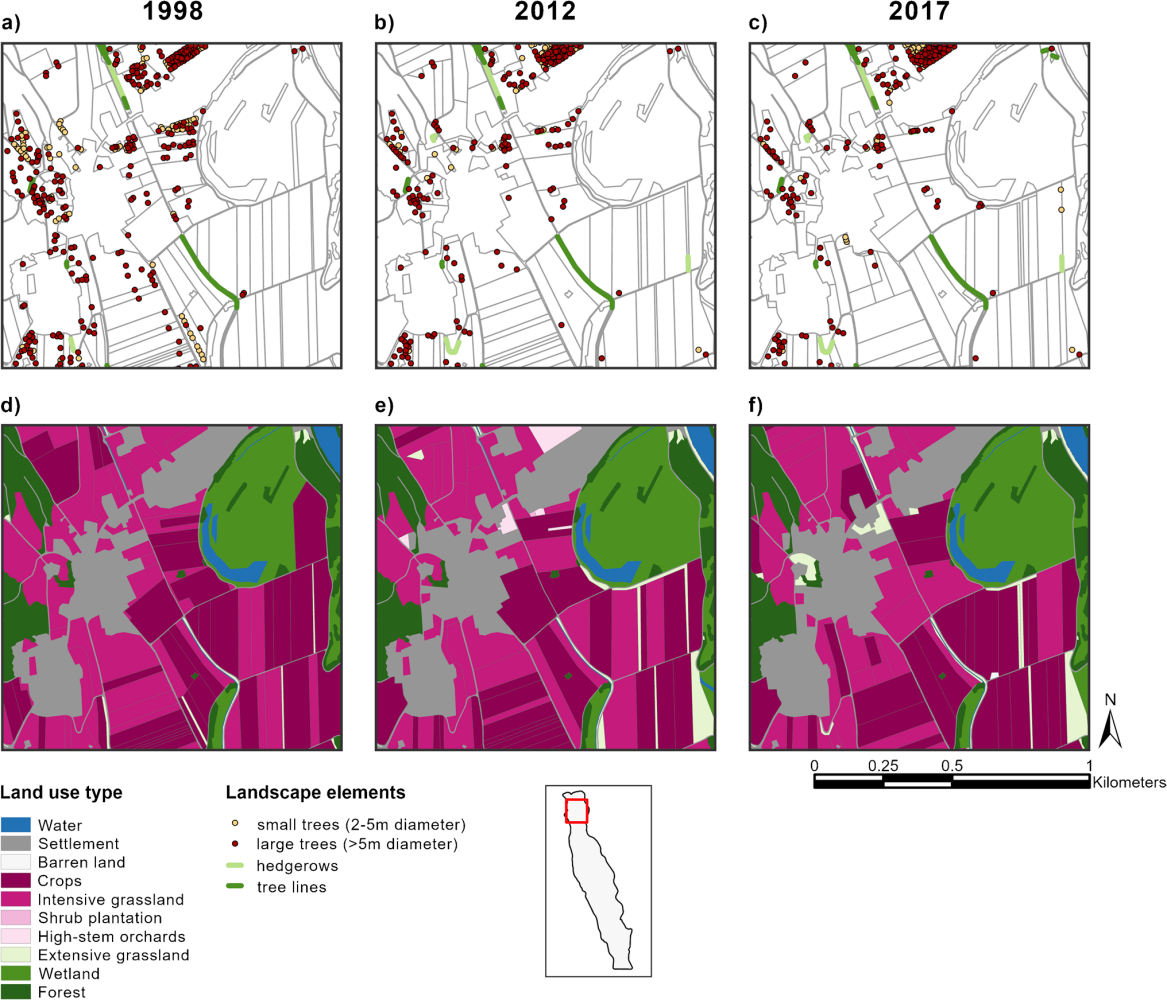
Example of landscape change in a representative part of the study area in Reuss, Switzerland. (**a**–**c)** field trees, hedgerows, and tree lines. Note the gradual disappearance of field trees. (**d**–**f)** Land use change. Note the expansion of the wetland area in the top right corner and the increasing field size. Also, note the increase in extensive grassland areas, which includes field margin vegetation and flower strips.

Landscape mapping showed a slight decrease in the total agricultural area by − 2.5% from 1781 to 1737 ha (Table 2). The decrease in the total agricultural area was mostly due to the conversion of intensive grassland and cropland to settlement (Fig. 3). The proportion of intensively used agricultural land also decreased as the share of extensive grassland grew, confirming the rise in ecological focus area reported in interviews. Meanwhile, the average field size increased from 1.35 to 1.81 ha (*p* < 0.0001) (Table 2), with a noticeable decrease in small (< 1 ha) crop fields (Supplementary Fig. 2). The total number of field trees decreased from 2229 to 1818 (− 18%), with large trees (− 23%) declining more than small trees (− 6%). These developments collectively describe land use rationalization through consolidation of larger fields and removal of field trees to facilitate management with large machines (Fig. 4).

Total semi-natural habitat area increased. While the area of high-stem orchards decreased (− 7.5 ha), extensive grasslands (+ 60.6 ha) and wetlands (+ 8.0 ha) increased in total area. Also, the total length of linear habitats increased by 11%, which was mostly due to the planting of new tree rows (+ 35%), while hedgerow length remained more or less stable (+ 2%). While old trees in high-stem orchards, relics of the traditional silvopastoral system, were scattered in the landscape, new trees were planted in lines mostly on field edges to facilitate agricultural management.

*Validation*

Variables that were determined both at the farm scale through interviews and at the landscape scale through mapping could be used for cross-validation of the two approaches. The 20 farms interviewed managed 724 ha, equivalent to 38% of the agriculturally used area in the case study landscape. Hence, the sum of farm-scale changes should be representative and reflect changes at the landscape scale. Indeed, the reported increase in ecological focus area was confirmed at the landscape scale, where we noticed an increase in flower strips, field margin vegetation, and other extensively used areas that are likely to qualify for agri-environmental scheme direct payments (Figs. 3 and 4). Many farmers also mentioned that they installed new semi-natural habitats in cooperation with a regional biodiversity conservation non-governmental organization due to financial incentives. Similarly, the sum of land uses determined by farmer interviews correlated well with mapping results (Supplementary Fig. 3). The fraction of agricultural area was 49% crop, 34% intensive grasslands, and 17% extensive grasslands according to interviews, and 45% crop, 37% intensive grasslands, and 18% extensive grasslands according to landscape mapping. Hence, the two approaches seem to be remarkably consistent for variables where consistency could be compared.

A second opportunity for validating observed changes is by comparing our results to a farmer survey carried out roughly 20 years ago in the same study area (Herzog et al. 2006). While this earlier farm survey had a different focus, questions relating to the farm area, crop diversity, livestock units, livestock density, N intensity on the main crop, and pesticide use on the main crop were similar enough to allow direct comparison. If we compare the median result from Herzog et al. (2006) with farmers reported values for 2000 and 2020 in this study, we see that median values of 2000 are closer to Herzog et al. (2006) than 2020 for farm area, crop diversity, livestock units, and pesticide use supporting the trends we report for those indicators (Supplementary Fig. 4). Livestock density had higher variability in our study than in Herzog et al. (2006), which can be explained by the fact that we interviewed a broader selection of farmers, including farmers with no livestock. Nitrogen intensity, on the other hand, was reported to be higher and more variable in Herzog et al. (2006) than by farmers interviewed in this study, which may be an indication that farmers in our study underestimated the former N intensity.

Our interpretation of the 1998 aerial photograph can be validated against habitat mapping in the field performed in parallel to the farmer survey mentioned above (Bailey et al. 2007). The 156.6 ha classified as wetlands in this study for 1998 is almost the same as 157.1 ha of base-rich fens, littoral zone of inland surface water bodies, seasonally wet and wet grasslands, and sedge and reed beds mapped in the field (data by Bailey et al. (2007), reanalyzed). Also, 32.8 ha of intensive orchards and high-stem orchards in this study are very similar to 34.3 ha fruit and nut orchards reported in the earlier study (Bailey et al. 2007). Comparison of these two land uses suggests high accuracy of aerial photograph interpretation, though we expect lower accuracy for the identification of extensive grassland, which was often difficult to differentiate from intensive grassland. For grasslands and other habitat types, the earlier study followed a different mapping protocol, so that validation is not possible.

### Desired change according to the three visions

All three stakeholder organizations want to improve agriculture to make it fitter for the future, and they all claim that their vision is sustainable. However, desired changes vary strongly between the three stakeholders and are oftentimes contradicting. There is not a single indicator for which all three visions agree on the desired direction of change (Table 3).

**Table.3:** Desired change and weight of indicators according to the three visions. Avenir Suisse is a liberal think-tank and promotes the opening of markets and a transition towards fewer, larger, more competitive farms. The Swiss Farmers Association represents a conservative force that wants to slow down change. The agroecological movement is represented by Landwirtschaft mit Zukunft, which supports smaller, more diversified farms with high levels of biodiversity.

Indicator	Avenir Suisse (AS)		Swiss Farmers Association (SBV)		Agroecological movement (LmZ)	
	Desired change	Weight	Desired change	Weight	Desired change	Weight
Farm-scale development						
Farm area	+ 1	1	0	1	− 1	2
Livestock units	+ 1	1	0	1	− 1	2
Crop diversity	− 1	1	0	1	+ 1	1
Livestock diversity	− 1	1	0	1	+ 1	1
Feed import	+ 1	2	− 1	1	− 1	2
Landscape-scale development						
Average field size	+ 1	1	0	1	− 1	1
Total agricultural area	− 1	1	+ 1	2	-	-
Proportion of intensively used agricultural land	+ 1	1	0	1	− 1	1
Social						
Farmer satisfaction	− 1/0/ + 1	1	+ 1	2	− 1/0/ + 1	1
Societal valuation	-	-	+ 1	2	+ 1	1
Fraction of farmers > 50 years old	-	-	+ 1	1	+ 1	1
Successor	-	-	+ 1	2	+ 1	1
Fraction of owned land	-	-	+ 1	1	+ 1	1
Economic						
Farm economic situation	− 1/0/ + 1	1	+ 1	2	− 1/0/ + 1	1
Price trend	− 1	2	0/ + 1	1	+ 1	1
Production trend	+ 1	2	+ 1	1	− 1	1
Off-farm work	-	-	− 1	1	− 1	1
Environmental						
Ecological focus area	0	1	0	1	+ 1	1
Semi-natural habitats	0	1	0	1	+ 1	2
N intensity	-	-	0	1	− 1	1
Pesticide use	− 1	1	0	1	− 1	2
Livestock density	-	-	0	1	− 1	1

AS represents market-liberal interests, desires, an increase in the farm area and livestock units, and a decrease in crop diversity and livestock diversity (Table 3). A key aspect of Avenir Suisse’s vision is promoting free trade so that agricultural products can be produced in countries/regions where they can be produced most efficiently. This implies higher feed imports since feed concentrates can be produced more cheaply abroad. In general, AS wants less land used for agriculture in Switzerland, since valorization by agriculture is much lower compared to other sectors. Other key elements of Avenir Suisse’s vision are decreasing prices for agricultural products and increasing production per farm, as well as decreasing direct payments to farmers. The focus is thus more on decreasing consumer prices and taxes in general. The vision does not formulate clear social goals for the farmer other than to reduce regulation of farmers to promote entrepreneurship and accelerate structural change towards fewer, larger, more specialized farms. We interpreted this to have ambivalent effects on farmer satisfaction and farm economic situations: while some farms may profit from reduced regulation, others would go out of business (Table 3).

The focus of the vision promoted by the Swiss Farmers Association (SBV) is on improving farmer economic and social situations (Monin et al. 2018). Key demands of the SBV are to improve the income and well-being of farmers, secure the family farm model for future generations, and improve the image of farmers in society (Table 3). For the most part, the SBV wants to slow down change and maintain current farm structures, agricultural landscapes, and levels of environmental protection. However, the SBV wants to increase the total area used for agriculture.

The agroecological movement (LmZ) is in many ways diametrically opposed to Avenir Suisse. LmZ wants smaller, more diversified farms operating in local value chains. Other key elements of their vision are a reduction in livestock units and livestock density, reduction in feed import, and improved biodiversity conservation (Table 3). This translates into more ecological focus areas, more and better connected semi-natural habitats, and reduced fertilizer and pesticide use (Kehnel et al. 2018). In terms of farmer satisfaction and farm economic situation, the desired changes of LmZ were interpreted to be ambivalent (akin to AS). While small farms may profit from these changes, larger farms, especially those that invested in intensive livestock production, would face severe difficulties.

**Fig. 5:**
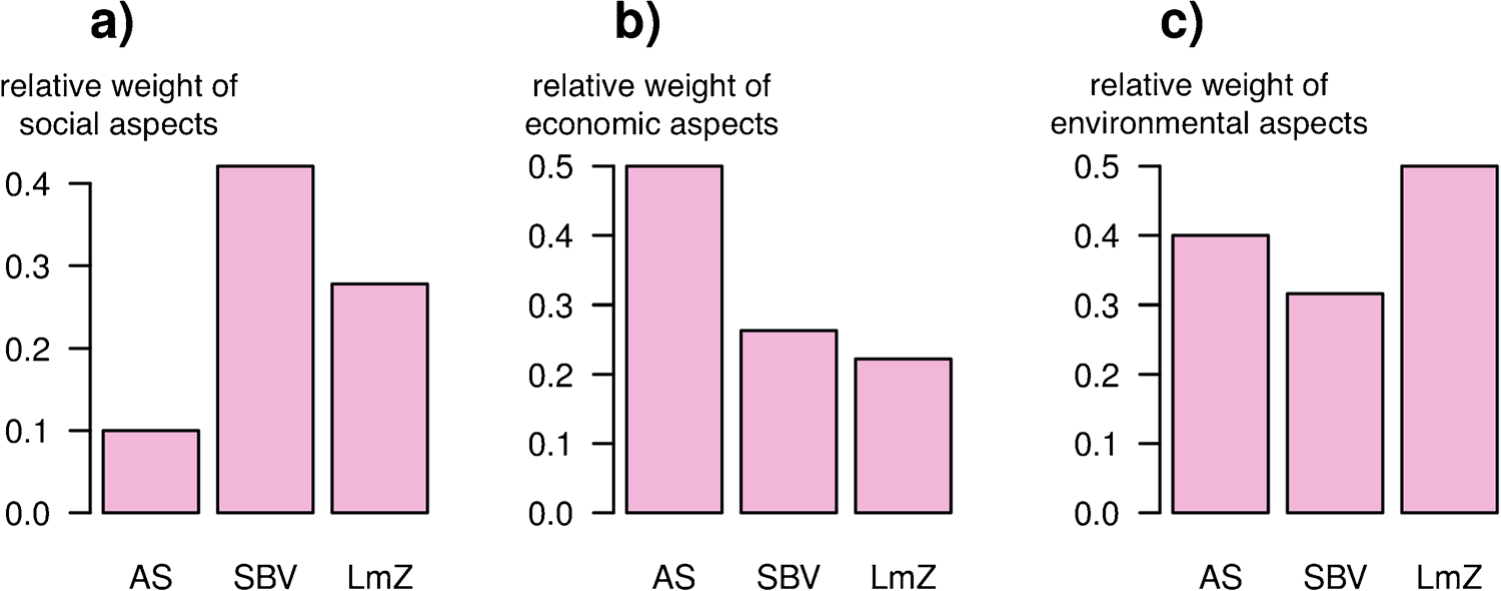
Relative sustainability focus of each vision. While the Swiss Farmer’s Association (SBV) prioritizes social sustainability aspects (**a**), Avenir Suisse (AS) prioritizes economic aspects (**b**), and the agroecological movement (LmZ) prioritizes environmental aspects (**c**). The plot was made by calculating the proportion of weights given to indicators from each sustainability dimension. A sensitivity analysis of the weights can be found in the supplement (Supplementary Fig. 4).

To check the consistency of the desired change matrix, we compared the sum of weights given to indicators from each sustainability dimension (Fig. 5). According to these weights, the Swiss Farmer’s Association (SBV) prioritizes social sustainability aspects such as farmer well-being, Avenir Suisse (AS) prioritizes economic aspects, and the agroecological movement (LmZ) prioritizes environmental aspects relative to the other sustainability dimensions. The focus of the visions on different aspects of sustainability is consistent with the political behavior of these groups. For example, the weights reflect the decreasing environmental focus from LmZ > AS > SBV (Metz et al. 2020) (Fig. 5c).

### Agreement between observed and desired development

**Fig. 6:**
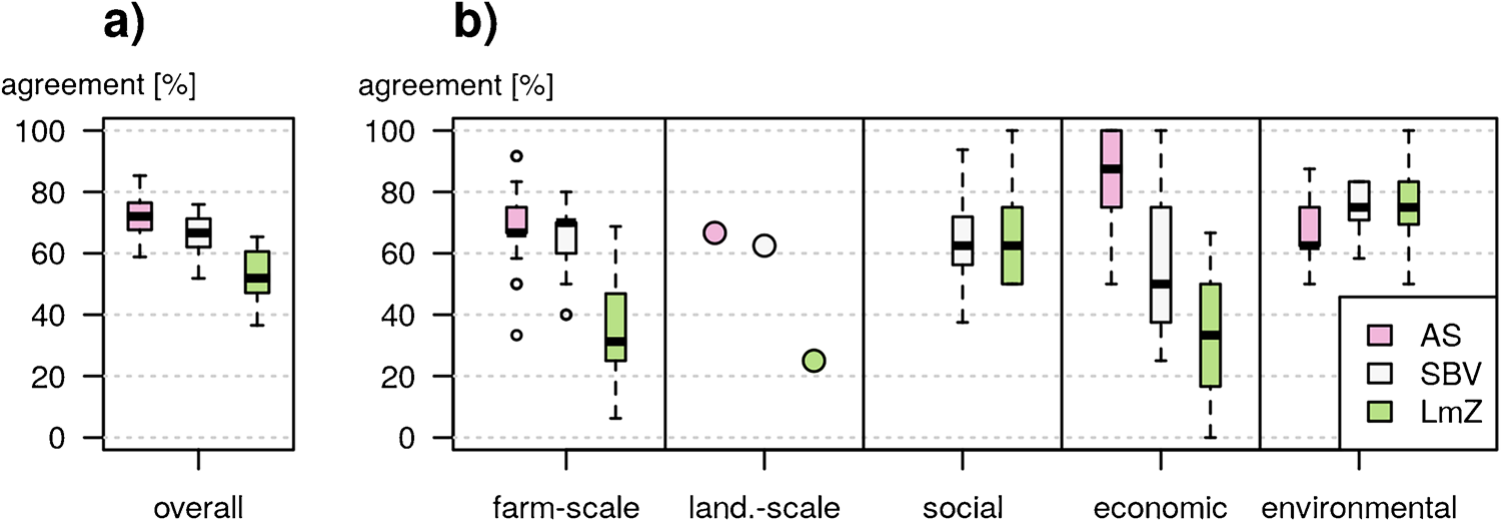
Agreement between observed and desired change. Agreement overall (**a**) and broken down into each of the five indicator categories (**b**). AS = Avenir Suisse, a liberal think tank representing free-market interests; SBV = the Swiss Farmers Association, representing a conservative force; and LmZ = Landwirtschaft mit Zukunft, an exponent of the Swiss agroecological movement. Agreement with indicators related to landscape-scale development is displayed as a single point for each vision. Agreement between observed and desired social indicators could not be determined for AS because this vision did not contain explicit social components.

The observed developments agreed most with the vision of AS. While the median agreement with AS was 72%, with SBV it was 67%, and with LmZ 52% (Fig. 6a). Agreement was significantly affected by the vision (Kruskal–Wallis test, *p* < 0.001), with significant differences between AS-LmZ (Dunn test, *p* < 0.001), SBV-LmZ (Dunn test, *p* < 0.001), but not between AS-SBV (*p* = 0.05). Although the median agreement between observed and desired development was highest for AS, there was considerable variability between the farms, reflecting their individual development trajectories. When looking at individual farm trajectories, small subsets of farms were more in line with the visions of SBV (25% of farms) and LmZ (5% of farms) as compared to AS (70% of farms). This suggests that while most farms developed in line with desired changes by AS, a few farms followed different trajectories. These farms not fitting into the main trend either had high levels of persistence and or focused more on shifting towards environmentally friendlier forms of production.

Breaking down total agreement into agreement by category revealed that agreement with AS was particularly high for farm structure and economic indicators (Fig. 6b). Most farms in the area increased in size and number of livestock units while becoming more specialized (Table 2), which reflects key elements of the AS vision. Also, productivity increased on almost all farms while prices received for farm products decreased for many farms (Table 2), so, for economic aspects, median agreement with AS was almost 90%. Contrary to the demands of AS, the overall budget for direct payments in Switzerland stayed constant over the period of study (Metz et al. 2020). Due to the contradictive nature of the LmZ and AS visions, LmZ had the lowest agreement in the categories where AS had the highest agreement (Fig. 6b). However, agreement was in general high with the environmental goals of LmZ, especially on some farms. This can be explained by the increase in biodiversity conservation efforts both at the farm and the landscape scale as a result of increased direct payments for such activities (Table 2) (Metz et al. 2020). However, other farms had little change in environmental indicators, translating to a high agreement with the SBV vision.

A sensitivity analysis revealed that the above-described agreement levels are fairly robust (Supplementary Fig. 5). In general, agreement between observed and desired change did not vary more than a few percentage points by varying the “no change” threshold from 0 to 10%. This is because most farm-scale changes were significantly stronger (Table 2, Supplementary Fig. 1). Similarly, the weights assigned in the desired change matrix (Table 3) also had little impact on the results. Setting all weights equally decreased agreement with AS (− 2.6%), increase agreement with SBV (+ 1.8%), and LmZ (+ 1.9%). However, neither changing the “no change” threshold, nor the weights, nor a combination of the above affected the rank of agreement between the visions (Supplementary Fig. 5).

### Ways ahead

In their review of progress in sustainability science, Sala et al. (2013) concluded that future development of sustainability assessment methodologies should focus on “holistic and system-wide approaches, the shift from multidisciplinarity toward transdisciplinarity; multiscale (temporal and geographical) perspectives, and better involvement and participation of stakeholders” (Sala et al. 2013). Progress has been made, and several new approaches adopted more multiscale and holistic sustainability framings. For example, Chopin et al. (2017) assessed cropping system sustainability in Guadeloupe from 2004 to 2010 by integrating indicators at the field, farm, and regional scale, while Barron et al. (2021) propose a system-wide and multiscale assessment of the sustainability of pasture-based dairy sheep systems including sustainability issues often omitted in earlier such assessments (Barron et al. 2021). However, as pointed out in a more recent review, there is still a gap when it comes to the involvement of stakeholders in the design of indicators and assessment of sustainability in agriculture (Chopin et al. 2021).

The main novelty of our approach is that it can accommodate multiple, even contradictory interpretations of sustainability, addressing the challenge of dealing with the normative dimension of sustainability assessment (Miller et al. 2014; Pascual et al. 2017; Schlaile et al. 2017). A recent study conducted workshops and interviews with stakeholders across Europe to define five socioeconomic pathways (SSPs) for agricultural development in Europe, including one sustainable pathway (Mitter et al. 2020). Such “what could be” scenarios implicitly represent the “what should be” visions of specific stakeholder groups. However, more often than not, stakeholders have opposing definitions of sustainable futures (Robinson et al. 2011; Zorondo-Rodríguez et al. 2014). By focusing on “what should be,” the approach presented here is aiming directly at stakeholder agreement/disagreement and not bothered by probabilities and internal logic of specific scenarios. In our Swiss case study, the three societal visions had almost diametrically opposing desires for future development: Avenir Suisse promoted “land sparing” and economic efficiency while the agroecological movement promoted more of a “land sharing” approach, focusing on different sustainability dimensions (Table 3, Fig. 5). This underlines that a legitimate sustainability assessment needs to be able to accommodate different visions as benchmarks for agricultural development rather than treating sustainability as an absolute value.

In addition to accommodating multiple interpretations of sustainability, our approach also meets the requirements of being holistic, transdisciplinary, and multiscale (see Sala et al. 2013). The approach (Fig. 2) is holistic since it covered cropping and livestock systems together, with indicators that are applicable to a wide set of farming contexts. Furthermore, by including questions on farmer satisfaction and societal valuation, we paid special attention to cover also social dimension of sustainability, which is chronically underrepresented in the agricultural sustainability debate (Janker and Mann 2018). Transdisciplinarity can be defined as crossing disciplinary and scientific/academic boundaries to develop integrated knowledge and theory among science and society (Tress et al. 2005). Our approach is a step in this direction, as it integrates scientific disciplines with non-academic knowledge contained in societal visions. Finally, our approach covers both the farm and the landscape spatial scales, and tracks the development over two decades, thus also including a temporal dimension. We learned that the temporal dimension was especially useful for comparing widely different farming systems, since focusing on the direction of change rather than absolute values smoothed out context variability between farms producing combinations of different crops and livestock. Also, analyzing change rather than one-time measurement removes the need of having (subjective) reference values inherent in most traditional sustainability assessment tools. The added value of analyzing both the landscape and the farm scale was that it allowed cross-validation (Sect. 3.3) and made it possible to detect that there is considerable variation in individual farm trajectories within the landscape.

While we screened visions from Swiss political interest groups, future work could utilize repositories such as the visions described by the Global Scenario Group (Electris et al. 2009; GSG 2021), which are applicable anywhere and resonate strongly with visions presented here. Alternatively, in future work, visions and indicators could be defined specifically for the case study region in stakeholder workshops (Mitter et al. 2019) or crowd sourced from citizens directly (Metzger et al. 2018). While being more resource and time intensive, such an approach would further strengthen the participatory element and increase the relevance of the vision for the case study area. Future studies should also consider that, like in all indicator-based assessments, the choice of indicators will influence the results (Kienast and Helfenstein 2016). In our case, the choice to focus on a broad set of indicators representing three sustainability dimensions was at the cost of not being able to analyze specific topics in more detail (such as farm economics of biodiversity loss). For indicators based on survey answers, an additional issue is that reported answers are biased by perception, which may lead to a distorted view of observed change. This bias can be accounted for by validating survey answers (where possible) with other data sources. Finally, it is important to be mindful of the possible consequences of outsourcing the normative aspect of sustainability assessments to societal visions. Visions may be heavily biased by partisan interests or may contain objectively unsustainable components. In our case, the AS vision only had a poorly developed social dimension in terms of the farmer, focusing more on lower prices and taxes for consumers, elements that the other visions did not prioritize (Fig. 5a). The fact that individual visions tend to focus on different aspects of sustainability, or for different stakeholder groups needs to be considered with required caution and transparency in such a comparison.

## Conclusions

In this study, we illustrated a novel approach to assess the sustainability of agricultural development based on contrasting societal visions in the Reuss valley, Switzerland. The assessment of one case study site alone, with all its peculiarities, does not allow drawing conclusions about the general development of Swiss agriculture and alignment with national visions. However, the case study example highlights the potential of societal visions to assess agricultural development as a foresight tool to improve regional governance. The analysis showed that agriculture in the study region underwent significant change, mostly following market-liberal forces. Bringing development of the region more on course with the vision of the Swiss Farmer Association would require stronger regulations that protect farmers from global market forces. Bringing development more on course with the vision of the agroecological movement would require a societal and political paradigm shift to smaller, more diversified farms with local food value chains and a stronger emphasis on environmental protection. From a methodological point of view, it was shown that accommodating multiple stakeholder goals makes normative decisions more transparent and results graspable for a wider public audience. For these reasons, we argue that future assessments of sustainability should be mindful of contrasting societal visions for agricultural development and try to explicitly account for these differences by comparing observed developments with desired change by various stakeholder groups. Convergence of stakeholder views is essential to progress from describing the system to taking action. By revealing stakeholder agreement for specific development indicators, the visions approach can identify low-hanging fruits for policy development.

## Supplementary Information

Below is the link to the electronic supplementary material.

Supplementary file1 (DOCX 1137 KB)

## Data Availability

The datasets generated during the current study are available from the corresponding author on reasonable request.
