# Supplementary material for: An approach for comparing agricultural development to societal visions
Source: Agron Sustain Dev. 2022 Jan 13;42(1):5. doi: 10.1007/s13593-021-00739-3 (PMC8758632; doi:10.1007/s13593-021-00739-3)
Supplement: Supplementary file 1 — Supplementary file1 (DOCX 1137 KB) [file 13593_2021_739_MOESM1_ESM.docx]

Contents

[Supplementary tables 2](#_Toc76547549)

[Supplementary Figures 5](#_Toc76547550)

[Questionnaire 9](#_Toc76547551)

[1. General questions 9](#_Toc76547552)

[2. Farm characteristics 9](#_Toc76547553)

[3. Farm economic situation 11](#_Toc76547554)

[4. Environmental aspects 11](#_Toc76547555)

[5. Social aspects 12](#_Toc76547556)

# Supplementary tables

Table 1. Habitat typology. Habitat types and descriptions are based on EUNIS habitat classification (EEA 2019).

| Habitat Type | Description | land use intensity | shape | minimum size and delimination | recognizable on aerial photograph? |
| --- | --- | --- | --- | --- | --- |
| water | includes all types of running and standing water bodies |  | polygon | 5x5 m. streams/ditches must be > 1 m wide. | yes |
| wetlands | wetlands, with the water table at or above ground level for at least half of the year, dominated by herbaceous or ericoid vegetation. | semi-natural | polygon | 5x5 m | Yes/no. wetlands recorded on topographic maps. |
| extensive GRASSland | extensive grassland and other extensively-used areas that are used (cut or grazed) < 3x per year. Includes flower strips, extensive meadows, field margin vegetation, and fallows. | extensive agricultural land / semi-natural | polygon | 5x5 m | Flower strips, field margin vegetation visible on aerial photographs. Fallows identified by comparing photographs from close by years. Conversation with farmers, locals, and field visits used to fine-tune differentiation between intensive and extensive grasslands. |
| intensive grassland | intensively used grassland > 3 uses (cuts or grazings) per year. May be either permanent grassland or rotational grassland. | intensive agricultural land | polygon | 5x5 m | See above. |
| shrub plantations | plantations of dwarf trees for non-food purpose. E.g. Christmas trees | extensive agricultural land / semi-natural | polygon | 5x5 m | yes |
| forest | woodland and recently cleared or burnt land where the dominant vegetation is, or was until very recently, trees with a canopy cover of at least 10%. | semi-natural | polygon | 10x10 m | yes |
| intensive orchards | stands of trees cultivated for fruit or flower production, providing permanent tree cover once mature. Only map as orchard if the trees are too small to be identified individually (< 2 m diameter). | intensive agricultural land | polygon | 10x10 m | yes |
| high-stem orchard | grasslands with > 20 field trees ha^-1^ and containing at least 3 field trees. | extensive agricultural land / semi-natural | polygon | - | Calculated after all areas were classified and all trees mapped. |
| settlements | buildings, industrial developments, transport network, waste dump sites. Include roads and railroads. |  | polygon | 5x5 m | yes |
| crops | annual crop production fields such as cereals that are not covered by grass | intensive agricultural land | polygon | 5x5 m | yes*. Use tractor tracks or clusters based on seasonal NDVI on aerial photographs to differ between young cereals and grasslands |
| barren land | rocky or other wise barren soil areas with less than 30% vegetation cover, usually construction sites |  | polygon | 5x5 m | yes |
| field trees | trees in agricultural land. Does not include tree rows. Since this is a point feature, this may overlay with other habtitats. Subdivided into small trees (2 – 5 m canopy diameter) and large trees (> 5 m canopy diameter) |  | point | > 2 m canopy diameter | yes |
| hedgerows | woody vegetation forming strips within a matrix of grassy or cultivated land or along roads, typically used for controlling livestock, marking boundaries or providing shelter. Hedgerows differ from lines of trees in being composed of shrub species, or if composed of tree species then being regularly cut to a height less than 5 m. |  | line | 40 m hedge length, gaps < 25 m (independent of field boundaries);  Hedgerows wider than 10 m to be attributed as woodland or scrubland. | yes |
| lines of trees | more or less continuous lines of trees forming strips within a matrix of grassy or cultivated land or along roads, typically used for shelter or shading. Lines of trees differ from hedgerows in being composed of species that can grow to at least 5 m in height and are not regularly cut down to a height below 5 m. |  | line | minimum of 3 trees which are less than 50 m apart | yes |

# Supplementary Figures


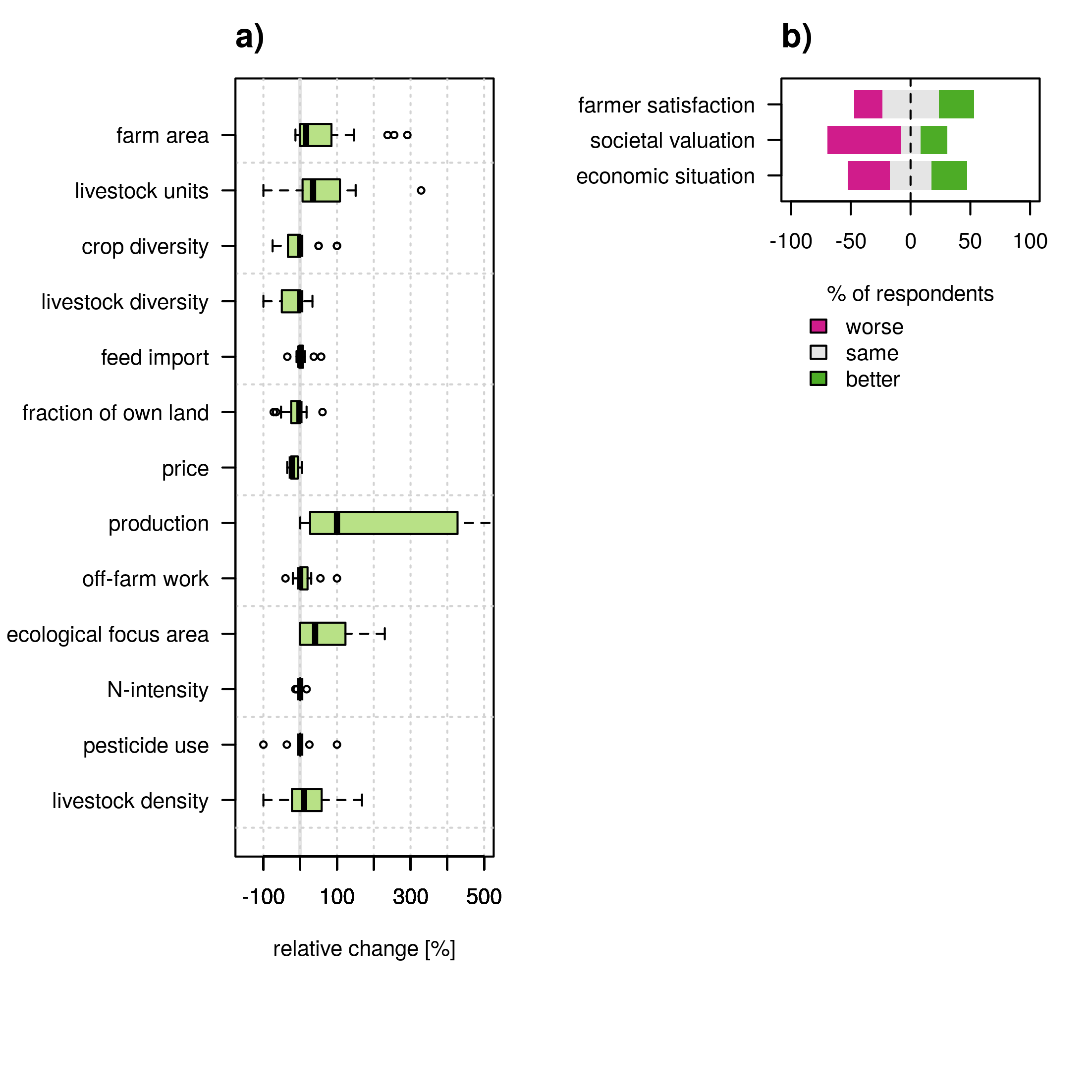


Figure 1. **Observed changes in farm-scale indicators.** The relative change for quantitative indicators (a). Perceived development in economic situation of the farm, farmer satisfaction, and societal valuation (b). All data based on farmer interviews (n = 20).


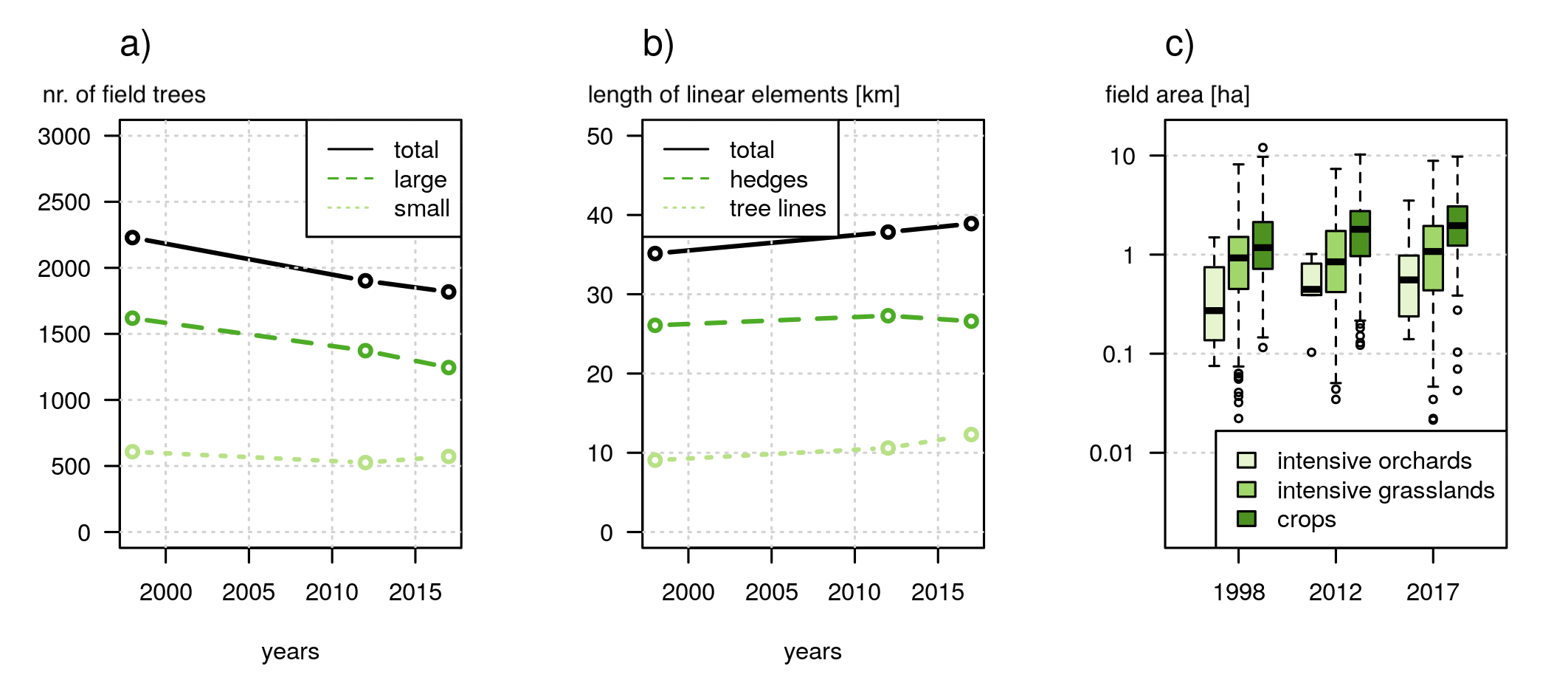


Figure 2. **Change in landscape structure between 1998 and 2017**. (a) field trees, (b) green linear elements, and (c) field area.


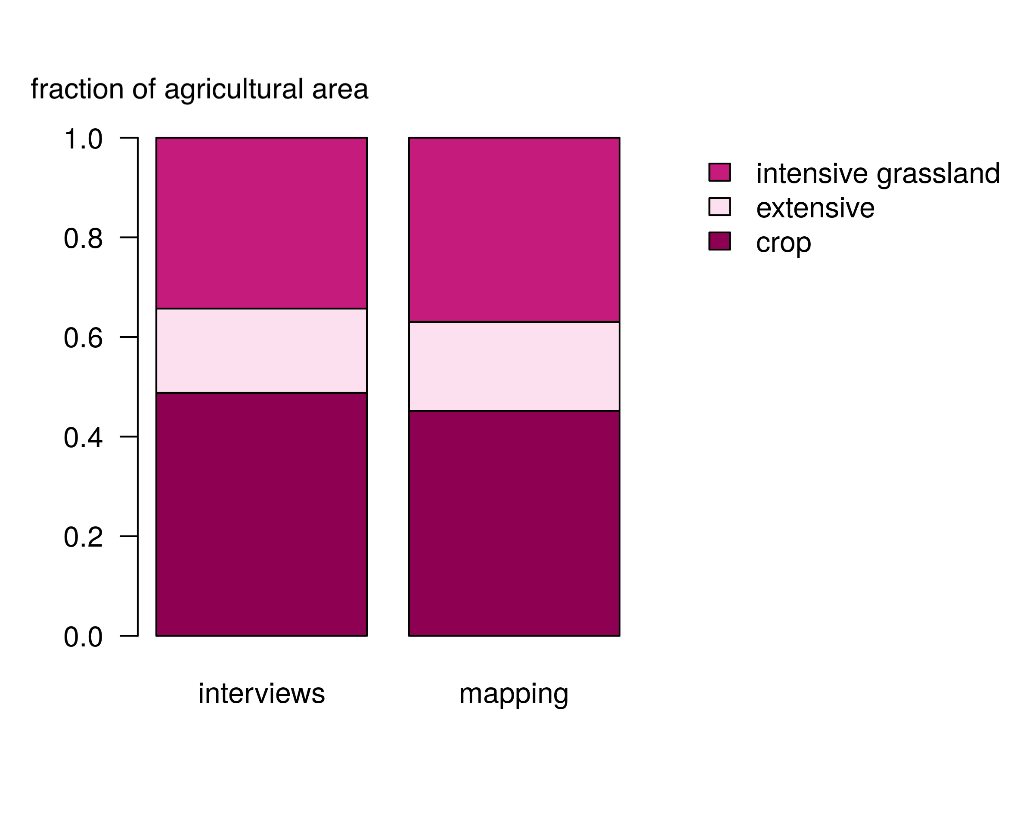


Figure 3. **Land use as determined by mapping and by farmer interviews**. Extensive includes wetlands, flower strips, and other extensively-used areas that are eligible for direct payments. Interview data based on 20 farmer interviews conducted in 2020 with farmers managing a total of 38% of agriculturally-used area in the case-study region. Mapping data based on Swiss Image orthophoto from 2017.


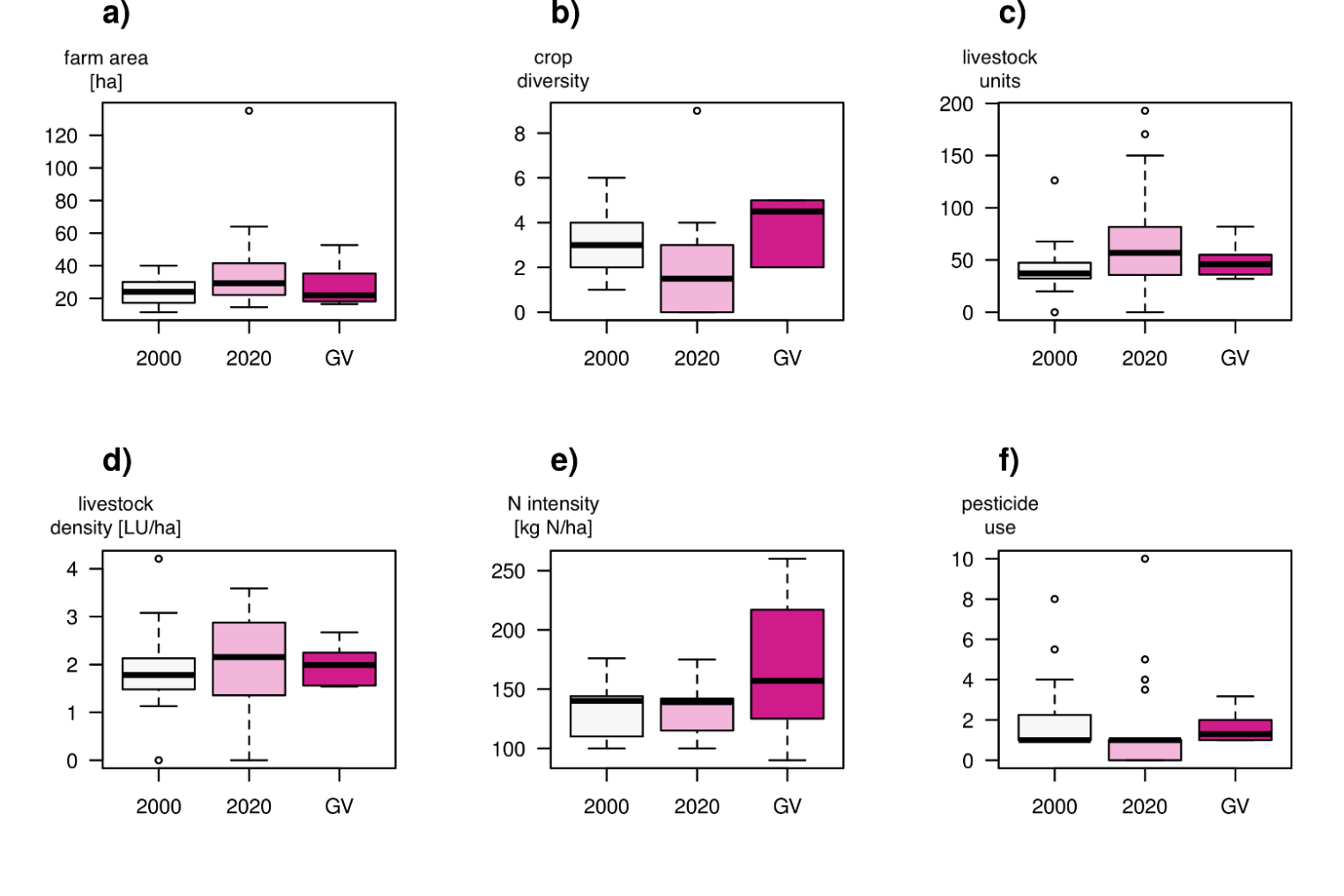


Figure 4. **Comparison to earlier study**. Farm area (a), crop diversity (b), livestock units (c), livestock density (d), N intensity (e), and pesticide use (f) according to farmer questionnaires conducted in 2002 (Herzog et al. 2006), farmers perceived status in 2000 if asked today, and status of today.


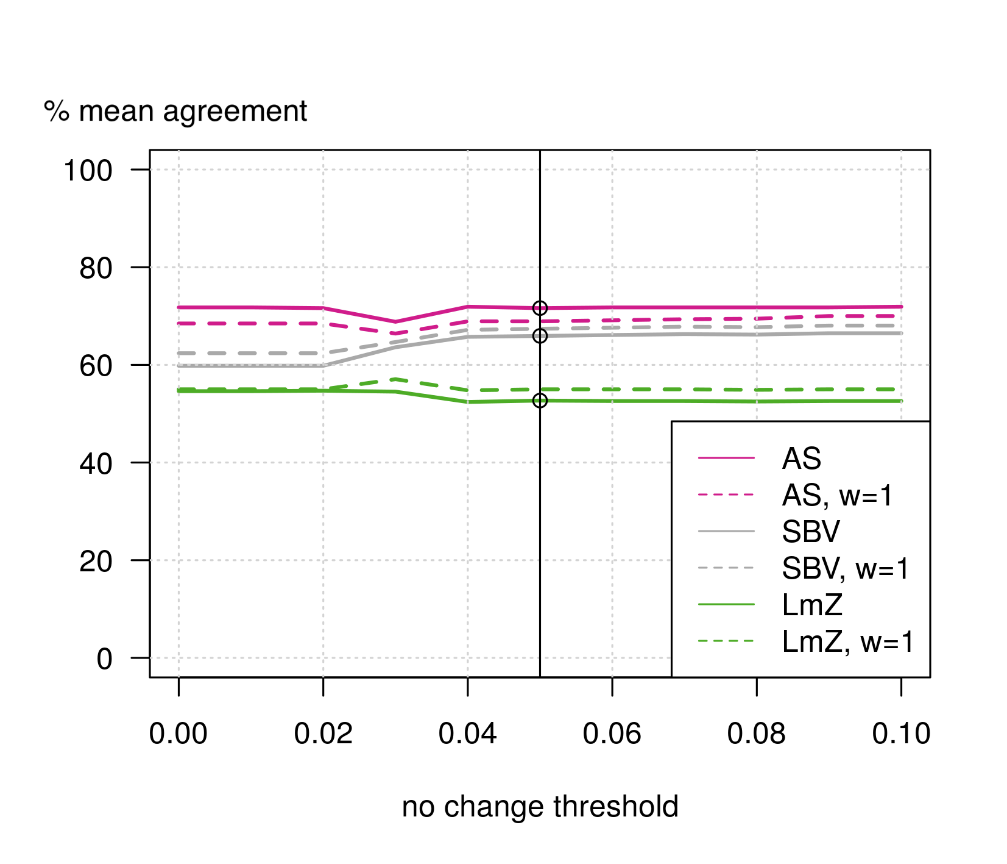


Figure 5. **Sensitivity analysis of calculated agreement**. Mean agreement is calculated as a function of the “no change” threshold. While full lines show agreement between desired change and observed change given the weights in the desired change matrix, the dashed lines show % agreement if all weights are set = 1. The black dots represent the values reported in the main text (no change threshold of 0.05).

# Questionnaire

**SIPATH:** Questionnaire on agricultural management intensity

**General information**

0.1 LTS Name: __________________________________________

0.2 Date: __________________________________________

0.3 Interviewer: __________________________________________

Farmer

0.4 Farm Number: __________________________________________

# 1. General questions

1.1. What type of farm do you have? *(Open question—record some keywords only)*

__________________________________________________________________________

__________________________________________________________________________

__________________________________________________________________________

1.1.1. Farm type: Arable farm Mixed farm Dairy farm Pig farm (other)_______

1.1.2. Business type: family farm corporate farm (other)_______

1.2 What is your age? ________________

1.3. Since when do you operate the farm? ________________

1.4. How much longer do you plan on operating the farm? ________________

1.5. Do you have a business (family) successor? ________________

1.6. Is the farm certified with labels? (e.g. organic) ❑ Yes ❑ No

1.6.1 If yes, which ones? _____________________________________________

1.7. What were the major changes to the farm in the past 20 years (if applicable)? *(Open question—record keywords only)*

__________________________________________________________________________

__________________________________________________________________________

__________________________________________________________________________

# 2. Farm characteristics

*Here and for all other indicators, for past state, best would be to get also a numerical answer. Second best is percent increase/decrease, or third best (minimum answer) just qualitative increase /decrease / stable /don’t know.*

**2.1. How much agricultural land is managed by the farm [ha]?**

total: ___________^2.1^ own land: ___________^2.1.1^ leased: ___________ ^2.1.2^

2.1.3. What was the agricultural area 20 years ago?

total: ___________^2.1.3^ own land: ___________^2.1.4^ leased: ___________ ^2.1.5^

**2.2. List land use of the farm and their area (share in the rotation).**

*Start with the crop which usually has the largest share in the rotation, then the 2^nd^ most important crop, etc.*

*The land use categories are defined to match the landscape mapping, so please follow the proposed format.*

| Crops in rotation | Name of crop | Area [ha] |
| --- | --- | --- |
| 1^st^ crop (largest area) | 2.2.1 | 2.2.1.1 |
| 2 ^nd^ crop | 2.2.2 | 2.2.2.1 |
| 3 | 2.2.3 | 2.2.3.1 |
| 4 | 2.2.4 | 2.2.4.1 |
| 5 | 2.2.5 | 2.2.5.1 |
| 6 | 2.2.6 | 2.2.6.1 |
| 7 | 2.2.7 | 2.2.7.1 |
| 8 | 2.2.8 | 2.2.8.1 |
| Total cropland (excluding rotational grassland) |  | 2.2.9 |
| Total intensive grassland (permanent and rotational grassland) |  | 2.2.10 |
| Total extensive grassland (ecological focus areas, low intensity meadows) |  | 2.2.11 |
| Permanent crop (such as orchards) |  | 2.2.12 |
| **Total UAA** |  | 2.2.13 |

**2.3. How many crops are cultivated?**

*Count the crops in the table, including permanent crops.*

________________

2.3.1. How many crops were grown 20 years ago? ________________

**2.4.** **List the type and number of livestock on the farm**

*Of interest here is a) the number of categories of livestock (LU) types held on the farm (degree of specialization), and b) the total livestock units. And how these indicators have changed over time. The table suggests LU conversion factors (Agridea 2019), but also local values can be used (1 cow = 1 LU). Other (smaller / younger) animals are counted and converted with factors to fertiliser LU in order to have a single measure for the animal density on the UAA.*

*For animals which are slaughtered when they are younger than one year (e.g. fattening chicken) the LU relate to the number of places available for this type of animals which are occupied throughout the year by several new generations of animals.*

|  |  |  |  |  |  |  |
| --- | --- | --- | --- | --- | --- | --- |
| Unit | Type of animal x | LU-Factor | Nr. animals | | LU units | |
|  |  |  | 2000 | Today | 2000 | Today |
| A | Milk cow | 1.0 |  |  |  |  |
| A | Suckler cow, nurse cow, bull | 1.0 |  |  |  |  |
| A | Calf (< 160 days) | 0.13 |  |  |  |  |
| A | Calf (160-365 days) | 0.33 |  |  |  |  |
| A | Young cattle 1-2 years | 0.4 |  |  |  |  |
| A | Young cattle > 2 years | 0.6 |  |  |  |  |
| A | Veal calf (calves raised on milk, slaughtered at ~4 months) | 0.04 |  |  |  |  |
| A | Horse and foal together | 1.0 |  |  |  |  |
| A | Other horses > 3 years | 0.7 |  |  |  |  |
| A | Foal 0.5 – 3 years | 0.5 |  |  |  |  |
| A | Pony, small horse, donkey | 0.25 |  |  |  |  |
| A | Milk goat | 0.2 |  |  |  |  |
| A | Other goats | 0.17 |  |  |  |  |
| A | Milk sheep | 0.25 |  |  |  |  |
| A | Other sheep | 0.17 |  |  |  |  |
| A | Lamb | 0.03 |  |  |  |  |
| P | Suckling sow (female pig with piglets) | 0.55 |  |  |  |  |
| P | Sow, non suckling (female pig alone) | 0.26 |  |  |  |  |
| P | Piglet 25 – 35 kg | 0.06 |  |  |  |  |
| P | Fattening pig | 0.17 |  |  |  |  |
| P | Pig for breeding (sow with piglets <30 kg) | 0.45 |  |  |  |  |
| P | Male pig for breeding | 0.225 |  |  |  |  |
| 100 P | Laying hen | 1.0 |  |  |  |  |
| 100 P | Young hen (2-2.5 circles/year) | 0.4 |  |  |  |  |
| 100 P | Broilers (fattening chicken, 6.5-7 circles/year) | 0.4 |  |  |  |  |
|  | other |  |  |  |  |  |
|  | other |  |  |  |  |  |
|  | SUM |  |  |  |  |  |

A: Individual Animal; P: Number of places occupied

**Degree of specialization in livestock**

*(Read the number of entries from table. For example, if the farm has 1000 pigs for breeding, 100 suckling sows and 5 males for breeding, the number of livestock categories would be 3.)*

2.4.1. Livestock categories 2000: ________________

2.4.2. Number of livestock categories today: ________________

**Number of livestock units**

*(Read the sum of livestock units from the table.)*

2.4.3. Total LU 2000: ________________

2.4.4. Total LU today: ________________

# 3. Farm economic situation

**3.1 How is the economic situation of the farm?**

| very difficult | |  |  | very good |
| --- | --- | --- | --- | --- |
| 1 | 2 | 3 | 4 | 5 |

3.1.1 How does the economic situation today compare to 20 years ago?

worse same better don’t know

**3.2. Are you also working off-farm? What percentage of your income is generated by off-farm employment?** *(If not working off-farm, write 0%)*

________________

3.2.1. What percentage of farm income was generated by off-farm employment 20 years ago (roughly)? ________________

**3.3. What is the most important product of the farm (e.g. milk)?** ________________

3.3.1. What percentage of farm revenue is generated by this food product (roughly, percentage)? ________________

3.3.2. To what degree has the amount of that product being produced changed since 2000? (± %)

________________

3.3.3. To what degree has the price being received for that product changed since 2000? (± %) ________________

# 4. Environmental aspects

4.1. What is the main crop by area (permanent or arable)?

Read from table 2.3

________________

**4.2. What quantity of nitrogen (kg/ha) do you apply to the main crop?**

^4.2.^____ kg/ha nitrogen per year *(typically this will range between 50 and 200 kg N / ha, depending on region and crop)*

4.2.1. What was the average N fertilization rate 20 years ago (also in kg N / ha)?

past N application rate: ^4.2.1.^________________

**4.3. What is the number of pesticide applications on the main crop?**

Total ___^4.3.1^ =  Herbicides ^4.3.1.1^___ + fungicides ^4.3.1.2^___ + insecticides ^4.3.1.3^___ + retardants ^4.3.1.4^___

What was the average pesticide application rate 20 years ago?

past application rate crop: ^4.3.2.^________________

**4.4. What is the origin of the animal feed?**

% from farm ^4.4.1.1^____ % from neighboring farms ^4.4.1.2^____ % retailer ^4.4.1.3^____

4.4.2. What was the share of local (farm and neighboring farms) animal feed 20 years ago? ________________

**4.5. What percentage of the farm is ecological focus area (EFA)?** ________________

*Cross-compliance requirements (usually 5%) and other landscape elements that are promoted by agri-environment schemes.*

4.5.1. What was the percentage of EFA 20 years ago? ________________

# 5. Social aspects

**5.1. How satisfying do you find your job on the farm?**

| not at all satisfying | |  |  | very satisfying |
| --- | --- | --- | --- | --- |
| 1 | 2 | 3 | 4 | 5 |

5.1.1. How satisfying is the job today compared to 20 years ago?

worse same better don’t know

**5.2. Do you feel like work on a farm as you are doing is valued by society?**

| not valued at all | |  |  | highly valued |
| --- | --- | --- | --- | --- |
| 1 | 2 | 3 | 4 | 5 |

5.2.1. How is societal valuation of your work today compared to 20 years ago?

worse same better don’t know
